# CCIO: A Cross-Chain Interoperability Approach for Consortium Blockchains Based on Oracle

**DOI:** 10.3390/s23041864

**Published:** 2023-02-07

**Authors:** Shaofei Lu, Jingru Pei, Renke Zhao, Xiaochen Yu, Xuyang Zhang, Junyi Li, Guanzhong Yang

**Affiliations:** 1 College of Computer Science and Electronic Engineering, Hunan University, Changsha 410082, China; 2 Hunan Provincial Key Laboratory of Blockchain Infrastructure and Application, Changsha 410082, China

**Keywords:** consortium blockchain, cross-chain interoperability, information sharing, blockchain oracle

## Abstract

Cross-chain interoperability can expand the ability of data interaction and value circulation between different blockchains, especially the value interaction and information sharing between industry consortium blockchains. However, some current public blockchain cross-chain technologies or data migration schemes between consortium blockchains need help to meet the consortium blockchain requirements for efficient two-way data interaction. The critical issue to solve in cross-chain technology is improving the efficiency of cross-chain exchange while ensuring the security of data transmission outside the consortium blockchain. In this article, we design a cross-chain architecture based on blockchain oracle technology. Then, we propose a bidirectional information cross-chain interaction approach (CCIO) based on the former architecture, we novelly improve three traditional blockchain oracle patterns, and we combine a mixture of symmetric and asymmetric keys to encrypt private information to ensure cross-chain data security. The experimental results demonstrate that the proposed CCIO approach can achieve efficient and secure two-way cross-chain data interactions and better meet the application needs of large-scale consortium blockchains.

## 1. Introduction

### 1.1. Background

Blockchains are an underlying core support technology that allow nodes in the network to jointly manage and supervise data through P2P networks, distributed consensus algorithms, and cryptographic methods [1]. Blockchains break the traditional relationship of relying on authorities to achieve trust and have attracted much attention due to their decentralization, transparency, non-repudiation, and traceability. A consortium blockchain is a collaborative community formed in a specific consortium context with a degree of openness between public and private blockchains. In recent years, with consortium blockchains being widely used in various scenarios, including finance, medicine, and other industries, there have been some increased complex issues about cross-chain interactions and information islands [2]. The consortium blockchain is a closed-world system that can only access data in the blockchain and does not have access to external information exchange. Therefore, the cross-chain interactions between different individuals and organizations deserve much attention.

The cross-chain interoperability refers to the effective communication and direct information exchange from one blockchain to another while preserving the essence of each blockchain, including its irreversibility and traceability [3]. Furthermore, the differences in technical standards, transmission protocols, and security mechanisms of consortium blockchains lead to difficulties in cross-chain interoperability [4,5]. The lack of third-party parsing and processing due to technical differences brings about difficulties in mutual recognition of data structures, and communication interactions cannot be carried out as required without uniform definition and processing due to different business models. Cross-chain interoperability can effectively expand the interaction and shared data of consortium businesses, and there are urgent requirements for mutual information interaction and value circulation. Furthermore, some current cross-chain technologies of public blockchains act as the circulation mechanism of the tokens, which results in that they cannot be directly applied to the inter-chain interactions of consortium blockchains that require high efficiency without tokens. Therefore, the following urgent problems still exist in the inter-chain interaction in consortium blockchains: the need to meet the high-efficiency interaction capability, the off-chain transmission security problem when interacting, and the cross-chain interaction format issue.

Blockchain oracles provide an attempt to solve the interaction between consortium blockchains. In blockchains, an oracle is essentially a trusted middleware that can actively collect off-chain data and feedback to the blockchain smart contract executor for on-chain and off-chain information invocation and access [6]. It can improve the closure to a certain extent, while the current blockchain oracle only provides a URL to access online data. There are also studies based on data migration oracles to achieve one-way migration of both consortium blockchains by default [7]. Still, the the default passive-triggered data migration approach is unsuitable for two-way efficient cross-chain interactions since there are validation problems and triggering processes for cross-chain requests in consortium blockchains. For proactive and efficient data interactions, improving efficiency and avoiding the risk of data exposure outside the consortium blockchains need to be urgently addressed.

### 1.2. Our Contributions

Previous cross-chain schemes have various limitations in solving the problem of data interaction of consortium blockchains. Most cross-chain approaches only apply to the token transfer of public blockchain rather than to the consortium blockchain of information interactions. In addition, the traditional blockchain oracle is limited to the URL. It cannot meet the requirements of cross-chain request verification, cross-chain parameter push, and results pull because the data of cross-chain requests cannot be obtained through the URL and need to interact with the destination blockchain. The previous one-way data migration scheme could not deal with illegal cross-chain requests, and timely data pulls had great passivity, which restricted the scalability of the consortium blockchain, reduced the efficiency of data interaction, and increased the interaction time. Therefore, focused on improving the efficiency of inter-chain interactions and ensuring standardized inter-chain interactions with safety, we propose CCIO for bidirectional cross-chain interactions in the context of the consortium blockchains. We improve the traditional blockchain oracle by adding a specialized smart contract to process cross-chain requests and novelly combining three oracle patterns: the pull-based outbound oracle, the push-based outbound oracle, and the push-based inbound oracle. In the cross-chain context, we combine a mixture of symmetric and asymmetric encryption algorithms with meeting the information interaction safety. To summarize, the main contributions of the approach are as follows:

1. We analyze the cross-chain information interaction architecture applied to the consortium business scenario and define the inter-chain interaction rules, including the consortium blockchain information mapping, identifying cross-chain service requests, and unified definition of cross-chain interaction formats;
2. Novel improvement of three blockchain oracle patterns to build a cross-chain oracle based on notary relay for automatic execution, optimizing and improving the pull-based outbound oracle from the perspective of data flow, and introducing a push-based inbound oracle and a push-based outbound oracle to meet the real-time and proactive nature of cross-chain information acquisition;
3. Considering the hidden danger of off-chain exposure of information in cross-chain interoperation, a mixed encryption method of symmetric and asymmetric keys is proposed and used to transmit the information of inter-chain interactions and ensure the security of cross-chain interoperation.

The rest of this paper is structured as follows. Related research work and the background literature on cross-chain interoperability is presented in Section 2. In Section 3, we describe the architecture and topology of CCIO. Furthermore, we present the proposed approach and the construction details in this paper. The experimental results and performance of CCIO are stated in Section 4. In Section 5, we discuss the results and how they perform from the perspective of previous studies and of the related areas. Finally, Section 6 concludes this article.

## 2. Related Work

Cross-chain interoperability can effectively expand the interaction capability and shared information of blockchain, and there have been many research works on blockchain interoperability technology. The existing blockchain, a closed-world system, cannot meet the requirements of the interactions in terms of scalability and differences. Many methods have been proposed to resolve these issues. Jian et al. [8] proposed a practical framework for data sharing among multiple blockchains that supported reliable data and controlled data access sharing by aggregating data from multiple blockchains and reorganizing them in the framework, where the authors regarded abstract smart contracts as a separate data module to implement hierarchical access control. The article [9] proposed a cross-chain scheme of agent networks to combat the shortcomings of current cross-chain technology in storing massive data. An agent cross-chain network was constructed by electing agent nodes in this paper. However, the method’s effect on blockchain applications needed reflection and demonstrated well in experiments. The authors in [10] defined a transaction-based asynchronous cross-chain exchange model with control conditions embedded to determine whether an asset transfer is performed, which was used to specify paired transactions and find eligible cross-chain transactions. However, the approach-based transaction dependency was specified for a public blockchain. The transaction structure needs to be changed to satisfy this method, which is costly and not universal. In [11], a cross-chain workflow model was proposed which defined the cross-chain process as a cross-chain workflow; it is an abstraction of cross-chain processes with similar characteristics, and the management of cross-chain workflows was realized with the relay chain as the centre. However, this approach required abstraction for detailed scenarios, and the abstraction model obtained for different cross-chain scenarios would have a large variability. Wu et al. [12] designed a cross-chain communication framework based on a periodic committee rotation mechanism, which connected different blockchains through committees with message-oriented authentication to improve the speed of cross-chain access. The committee rotation mechanism improved the scalability of the blockchain but also introduced the complexity of the election of members, which took a large part of the entire cross-chain access process. The article [13] proposed a cross-chain communication method with a simplified node communication topology mechanism, defined the construction rules for node identity path proofs, and dynamically constructed and verified cross-chain transaction path proofs. More attention should have been paid to the loyalty of nodes and a poor focus on avoiding security risks of transmission outside blockchains. The authors in [14] described a cross-chain exchange-based management system to improve the scalability of blockchain applications in an onboard self-organizing network by introducing public key encryption with a keyword search.

There are also an increasing number of research works on the blockchain oracle, but most of them are used for on-chain contracts to obtain data outside the blockchain through traditional URLs. Some enlightening articles suggest that blockchain oracles are used for inter-chain interactions. Albreiki et al. [15] described the role of the oracle in the blockchain system; it connects data from the outside world to the blockchain in a centralized and distributed environment. Its workflows are typically executed among three types of participants: data providers, oracle components, and blockchain operators. The data feedback program reads data from various online data sources through different web APIs and communication interfaces, which then applys authentication mechanisms to transmit highly accurate, relevant, and reliable data to the blockchain system. Mühlberger et al. [16] analyzed and discussed a blockchain oracle used for on-chain and off-chain data transmission through two core dimensions of transmission direction and the origin of the data stream. A practical application of the Internet of Things was constructed to illustrate the effect of improving blockchain closure for the blockchain oracle in this paper. However, the blockchain oracle described in this paper is just traditional, which could only be used to obtain data outside the blockchain and could not be directly applied to cross-chain data interaction scenarios. In [7], a cross-chain data migration architecture was constructed based on a data migration oracle in the context of blockchain data migration. A data migration mechanism was designed that was similar to opening a secure channel between two blockchains to allow secure data migration. This mechanism brought a good idea of interaction. However, it only applies to the default data migration between two blockchains and cannot verify the legitimacy of cross-chain requests. It required an artificial trigger action to make it impossible to obtain requests and transmit data in time, which did not meet the high efficiency of data interaction ability. Besides, there is only one ideal method in this paper, and no experiment can verify the actual migration effect.

According to the aforementioned derivation, the efficiency of information interactions between consortium blockchains and the security of off-chain transmission are urgent problems to be solved in cross-chain technology. The concept of notary relays can solve the problem of the unified definition of data interaction. However, the trust of a notary in the cross-chain of public blockchain assets is usually embodied as the authority department, which is not fit for the data interaction of the consortium blockchains. In this case, a suitable trust mechanism is needed. Currently, the traditional blockchain oracle only provides network resource access to obtain online data. In this case, it needs to specify the network URL to pull the off-chain data. In addition, few studies have applied it to the two-way interoperation scenario of consortium blockchains, which require efficient interaction. That inspires us with a feasible idea: reasonably improving the blockchain oracle can find a better solution for efficient inter-chain interoperation of consortium blockchains.

## 3. Materials and Methods

### 3.1. The Proposed Cross-Chain Architecture

**Figure 1:**
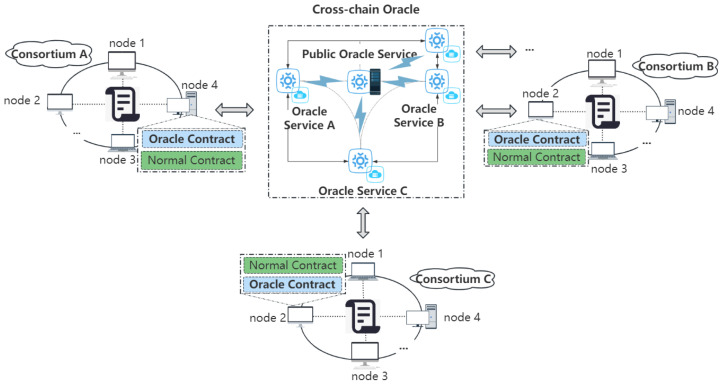
The overall architecture of CCIO.

In this section, we present the architecture of CCIO for consortium blockchains, as shown in Figure 1. A traditional oracle breaks the closure of the blockchain and the idea of notary relay in public blockchain cross-chain technology makes interactions simple, which is the inspiration for our proposal of a CCIO architecture. To optimize the construction of the cross-chain oracle by transforming the traditional oracle, the oracle plays the role of notary relay, serving as a cross-chain without human interference, so we put forward the architecture of CCIO. CCIO consists of two main modules: the consortium blockchains participating in cross-chain and the efficient cross-chain oracle based on the notary relay. The former are consortium blockchains with cross-chain demand, including the source consortium blockchain representing the request sender and the destination consortium blockchain where the data is located, which are equipped with a complete consensus algorithm and appropriate nodes, respectively. The latter function as a cross-chain relay trusted notary, which is improved and constructed by a variety of traditional oracle patterns. We will then elaborate on the role of the two modules of the CCIO architecture and how this design solves our problem.

#### 3.1.1. The Consortium Blockchains Participating in Cross-Chain

As shown in the periphery in Figure 1, each consortium blockchain can be served as a request blockchain to send cross-chain requests and can also be used as a data source blockchain to provide on-chain information for other requests. Therefore, cross-chain requests are bidirectional. Data can flow two ways between authorized consortium blockchains, and once a user initiates a cross-chain request, there is no human manipulation involved. Our scheme automatically queries the destination consortium blockchain to obtain information for cross-chain request feedback. Among them, the two types of smart contracts that need to be deployed are common smart contracts and oracle smart contracts. The common smart contract is mainly used for business processing on the consortium blockchain. As an on-chain component, the oracle smart contract interacts with the off-chain pegged oracle service. It is used to collect and process the cross-chain requests initiated by the users, query the received request parameters, and store the returned results.

In addition to deploying the oracle smart contract and providing access, the consortium blockchain participating in the cross-chain can deploy the nodes and handle the business logic according to their own business needs without the need for separate design to accommodate the cross-chain scheme.

#### 3.1.2. An Efficient Cross-Chain Oracle Based on Notary Relay

A blockchain oracle acts as a trusted middleware to transfer information from the off-chain world to the blockchain and serves as a bridge for on-chain and off-chain interactions [15,17]. Based on the interaction type, blockchain oracles can be classified as inbound oracles that insert data from the external world into the blockchain or outbound oracles that allow smart contracts to deliver data to the external world [17,18]. Based on the direction of data flow, the initiator of data flow, and whether it is a push or pull-based communication, there are four combinations of these options. Current research on blockchain oracles is mostly based on on-chain and off-chain interactions, and few researchers have considered its combination with cross-chain scenarios.

The proposed architecture of CCIO is the novel combination and design of an efficient cross-chain oracle by improving multiple traditional blockchain oracle interaction patterns. In the context of a consortium blockchain, the cross-chain oracle transmits data as an automatic execution notary relay. As shown in Figure 1, the oracle services in this architecture are mainly divided into two categories: the oracle service of pegging contracts and the public off-chain oracle service.

(1) The Oracle Service of Pegging Contract
  The specific off-chain oracle services are pegged by deploying oracle smart contracts on the consortium blockchain participating in the cross-chain. The off-chain oracle service is mainly used to obtain cross-chain requests immediately, obtain query parameters, and receive the queried results, to complete the interoperation between the data request consortium blockchain and the consortium blockchain where the data resides.
(2) The Public Off-chain Oracle Service
  The public off-chain oracle service functions as a forwarding and verification station. It uses predefined rules for interaction between consortium blockchains, including the mapping of each consortium blockchain participating in cross-chaining, cross-chain request parsing format, encryption and decryption rules for requests and data, and consortium blockchains specified forwarding rules to validate cross-chain requests, collect service information, and perform forwarding of services.

Taking a specific cross-chain request as an example; firstly, when a user sends a cross-chain request from one consortium blockchain, it is processed by the oracle service of pegging its contract. According to the predefined rules for inter-chain data interaction between consortium blockchains, after verification by the public off-chain oracle service, the oracle service pegged by the consortium blockchain where the data is located conducts inter-chain interactions. It then receives the results, thus completing a cross-chain interoperability process. Moreover, considering that different users in each consortium blockchain may send the same cross-chain request, to avoid unnecessary time and resource overheads caused by repeated requests, our solution stores partial data, such as the transaction hash of those requests, while returning the results, which also facilitates the traceability of the cross-chain process. In addition, because cross-chain interoperation is often uncertain between the specified consortium blockchains, our architecture is also suitable for multi-chain interactions.

### 3.2. The Proposed Cross-Chain Method

In this paper, we propose CCIO for inter-chain interactions, which consists of three main components: interaction rules, design of the efficient cross-chain oracle, and security guarantee of data transmission. When a cross-chain request is emitted for cross-chain data interaction, the predefined interaction rules are bound to follow. Then, the cross-chain oracle component forwards the cross-chain request to the pegged off-chain target oracle services, which queries and stores the data in the consortium blockchain where the data is located. At the same time, to avoid security risks during data transmission, the oracle service can selectively encrypt private data during transmission.

#### 3.2.1. Interaction Rules

Data between consortium blockchains need to be uniformly defined and interacted with according to rules, even between homogeneous consortium blockchains. When a new consortium blockchain participates in the cross-chain process, it must follow predefined mapping and interaction rules to carry out the cross-chain process. The oracle component needs to resolve and forward the cross-chain requests and specify the next forwarding services according to the rules. The inter-chain interaction rules in this scheme mainly include cross-chain service mapping and identification, the forwardable scope of oracle services, service registration and discovery, and data interaction format.

- Cross-chain Service Mapping and Identification

In our method, consortium blockchains participating in cross-chains should have different service identifiers according to their description information, to facilitate the designation and registration of cross-chain services in the public oracle service. The cross-chain services are identified here as *vId*:

(1) vId=<chainInfo,description>,

the *vId* is the availability for registering with the specified service, where *chainInfo* is the information of the consortium blockchain represented by the *vId*, and *description* is the information of consortium blockchain. The cross-chain service identifier *vId* is bound to the consortium blockchain and represents the inter-chain interactions of the blockchain. When the *vId* is included in the request information, it means that the request is a cross-chain request.

- Forwardable Scope of Oracle Services

This refers to the consortium blockchain information participating in the cross-chain and service information that can obtain data from this blockchain. Therefore, each consortium blockchain has the following service forwarding mapping *chain_scale*:

(2) $$chain\_scale=<vId_{i}\ldots \ldots vId_{n}>$$

where $vId_{i}$ is the cross-chain service identifier. After receiving a cross-chain request, the public oracle service searches for the requesting blockchain and the cross-chain service in the *chain_scale* of the destination blockchain for verification.

- Service Registration and Service Discovery

Combined with the current service, the registration, and the discovery techniques of the SOA, the public oracle service will perform registration for the participating consortium blockchains and cross-chain services based on the description information and identification. The public oracle can specify the service and run service discovery programs by the blockchain description information or the indicated *vId* carried in the cross-chain request.

- Data Interaction Format
  The predefined data interaction formats mainly include cross-chain request formats and service forwarding formats.
  (1) Cross-chain Request Format
  The cross-chain request is processed by the oracle smart contract for a transaction initiated from the consortium blockchain or invoked by a common smart contract on the consortium blockchain, and its format is as follows:
  (3) reqInfo=<vId,to_vId,to_chain,from_account,extra,tx_hash,encryptToTRes>
  where *vId* is the cross-chain service identifier, indicating the service initiator and requesting consortium blockchain information; *to_vId* represents the destination service; and *to_chain* is the target consortium blockchain description information. Note that at least one of the two fields here is non-empty. When the requestor cannot identify the destination service identifier *to_vId*, the public oracle service performs service discovery through *to_chain*. *from_account* is the account that sent the cross-chain request, the *extra* field describes the additional description information, *tx_hash* represents the transaction hash of the cross-chain request, and *encryptToTRes* is the ciphertext encrypted with the public key of the public oracle service.
  (2) Service Forwarding Format
  Service forwarding helps to find the target cross-chain service designated by the public oracle service for the requesting party. It is used to forward the request information to the destination cross-chain service for parsing and processing. The format is as follows:
  (4) transInfo=<vId,to_vId,from_account,extra,tx_hash,resX,encryptToXRes>
  Among them, in addition to the mentioned *vId*, *to_vId*, *from_account*, *extra*, and *tx_hash* fields, the service forwarding format adds the *resX* field, which is the specified service result generated by the public oracle service after verification of *reqInfo*, including the verification result about *to_vId* and the *encryptToXRes* field (the ciphertext of cross-chain service *X* public key encryption).

#### 3.2.2. Design of the Efficient Cross-Chain Oracle

**Figure 2:**
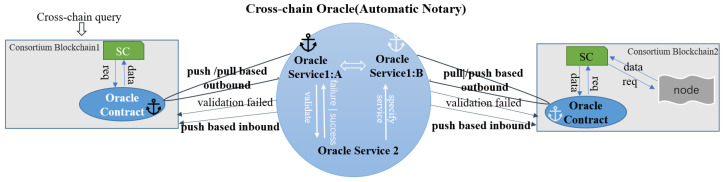
Inter-chain interaction flow.

In the consortium blockchain context, the cross-chain oracle transmits data as an automatic execution notary relay. It pegs the specific oracle service by accessing the oracle smart contract to complete the inter-chain interoperation between the data-requesting consortium blockchain and the destination consortium blockchain. Figure 2 shows the detailed inter-chain interaction process. The core cross-chain oracle for efficient data interaction between consortium blockchains is divided into three modules: the pegging of oracle smart contracts and specific oracle off-chain services of both consortium blockchains, the interactions of oracle smart contracts and oracle services, and the operations of the public oracle service.

(1) Pegging of Oracle Smart Contracts and Off-chain Services

It is necessary for consortium blockchains involved in cross-chain interoperation to function for their business scenarios and deploy normal smart contracts that handle business logic. Furthermore, both consortium blockchains need to deploy oracle smart contracts serving cross-chain processes.

The oracle smart contract is the on-chain artefact which anchors the off-chain oracle services; when an oracle smart contract is deployed to a consortium blockchain, a contract address is returned in the receipt of the deployment transaction. In combination with the identity mapping of the consortium blockchain involved in the cross-chain process, the off-chain oracle service of the consortium blockchain can be uniquely specified and established. Algorithm 1 shows the core method descriptions and event definitions of an oracle smart contract which processes cross-chain requests initiated by users of this consortium blockchain. Firstly, the oracle smart contract verifies the legitimacy of cross-chain request parameters and specifies the logical steps when the verification succeeds or fails. Next, it parses the received request parameters, invoking the relevant smart contract on the consortium blockchain and entering the query process of the data on the consortium blockchain. It is necessary to trigger the related log events. Then, the oracle smart contract interacts with its pegged off-chain oracle service and returns the query result data to the cross-chain request side. While the contract triggers and records the result feedback log, the transaction hash and the part of the data with identification contained in the result data set are stored to avoid the waste of resources if there are the same cross-chain requests.

**Algorithm 1:** The Logic of *OracleSC*

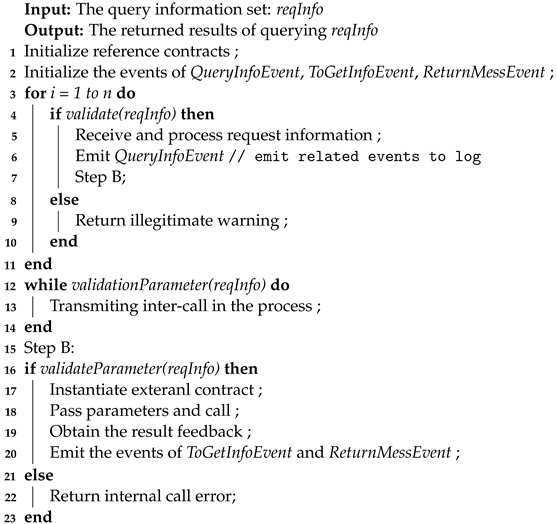

**Figure 3:**
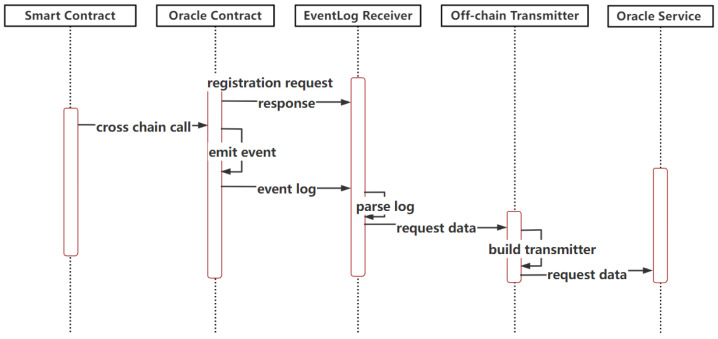
Push-based outbound oracle.

**Figure 4:**
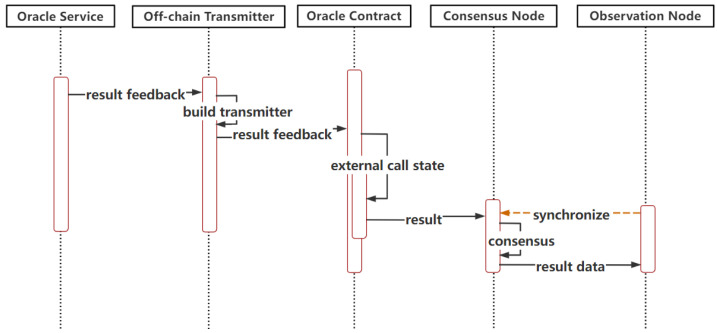
Push-based inbound oracle.

**Figure 5:**
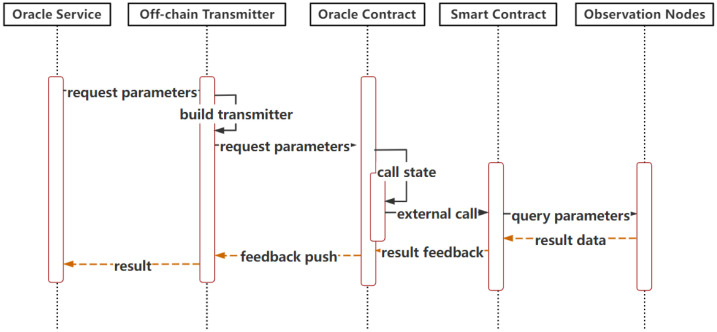
Pull-based outbound oracle.

(2) Interactions of Oracle Smart Contracts and Oracle Services
  To meet the requirements of efficient data interaction between consortium blockchains, we optimized the oracle patterns from the perspective of the data flow direction and introduced oracle smart contracts to process requests in a variety of traditional blockchain oracle patterns, including push-based outbound oracle, push-based inbound oracle, and pull-based outbound oracle. Unlike the data migration background in article [7], when one-way data are migrated across consortium blockchains, the pull-based inbound oracle requires active human control of the trigger timing. That is only applicable to the migration of both definite blockchains and cannot satisfy the two-way data transmitted across different consortium blockchains due to the exact demands of timeliness and automaticity. For CCIO, after the user initiates a cross-chain request, the cross-chain process will be completed automatically on time without active human intervention. The cross-chain process mainly involves three varieties of blockchain oracle patterns we proposed, which were designed for bidirectional cross-chain interactions and introduced a specialized oracle smart contract to handle the cross-chain request by us based on a traditional oracle. These blockchain patterns were the push-based outbound oracle, the push-based inbound oracle, and the pull-based outbound oracle. Furthermore, we combined them in a novel way to build an efficient cross-chain oracle.
  - Push-based Outbound Oracle in the Cross-chain Process
    As shown in Figure 3, when the oracle smart contract on the consortium blockchain is invoked by users or other smart contracts, it will trigger internal calls and will write to the event log composed for each topic. The event log receiver records the event invocation information by subscribing to the topics of cross-chain requests, which are filtered and parsed to obtain the cross-chain data. Once the cross-chain data are found, the oracle smart contract immediately interacts with the oracle service and starts the subsequent process to meet the timeliness of cross-chain request processing.
  - Push-based Inbound Oracle in the Cross-chain Process
    Figure 4 depicts the sequences of this process. When the oracle off-chain service receives the result feedback of a cross-chain request sent by a user, once it is verified that the required data are obtained, the results are immediately pushed into the pegged consortium blockchain via off-chain transmission. The pegged oracle smart contract feeds the obtained results to the consensus nodes after the external invocation of the smart contract and storage states. After finishing the consensus process, the results are stored and synchronized to other nodes through the P2P network. The push-based inbound oracle enters the subsequent process simultaneously to meet the timeliness of the data received.
  - Pull-based Outbound Oracle in the Cross-chain Process
    As shown in Figure 5, when the oracle service receives the request parameters to query the data of the currently pegged consortium blockchain, it will actively interact with the on-chain component to pass the query parameters. The oracle smart contract invokes and obtains the states to gather the required data from the nodes. When the required data are obtained, the common smart contract returns to interact with the off-chain transmission component and pulls the data out to the oracle off-chain service, where the service parses and combines the data according to the interactive format. The subsequent processes meet the initiative of querying data.
(3) Operations of the Public Oracle Service

The public oracle service mainly verifies whether the identity of the inter-chain data interoperation requestor is credible and legitimate and whether the requested data conforms to the specification. Figure 2 shows the inter-chain interaction process. The public oracle service first verifies the inter-chain requests, and those that fail to pass the verification will directly give failure feedback on the inter-chain to avoid consuming unnecessary inter-chain communication costs. The public oracle service collects information and forwards services for the verified inter-chain requests through the predefined consortium inter-chain interaction rules. The inter-chain interaction rules mainly include the mapping information of each consortium blockchain, the parsing format of inter-chain requests, the encryption and decryption rules of requests and data, and the forwarding rules. Finally, the parsed and combined information is transmitted to the oracle service pegged to the consortium blockchain.

#### 3.2.3. Security Guarantee of Data Transmission

The internet differs from LAN in that cross-chain interactions may expose information to the off-chain world or be maliciously hijacked during transmission. Secure encryption methods must be used for the necessary private data to avoid security risks in the off-chain world and during transmission. Blockchain oracle is an essential and trusted middleware for access to blockchain and off-chain data. Currently, the trustworthiness of a blockchain oracle is mainly supported by a trusted execution environment or around TLS supplemented with cryptography, including TLS-Notary protocol, TLS-N protocol, etc. [15,19]. Here, we introduce a mixed encryption method combining symmetric and asymmetric keys to secure data with the help of the trusted foundation of a blockchain oracle.

Our method mainly generates asymmetric keys locally in the trusted oracle, distributes securely encrypted symmetric cyphers, and then selectively encrypts private data to avoid security risks in the off-chain world. The key is considered to be generated locally in the oracle because neither the public blockchain nor the consortium blockchain is suitable for complex computation on the blockchain, and the computational overhead of encrypting and decrypting data on the blockchain is enormous. Moreover, asymmetric public key encryption is limited by data capacity, and the speed becomes slower with the increasing volume.

**Figure 6:**
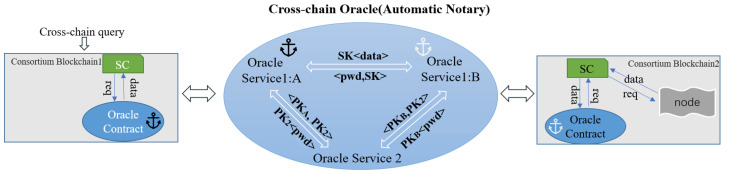
Mixed encryption for off-chain data.

As shown in Figure 6, each oracle service undergoes a complete communication interaction when it generates or updates its asymmetric key, *<PK, PR>*, locally. *Oracle Service 1:A* exchanges *<$PK_{A}$, $PK_{2}$>* with *Oracle Service 2* (public oracle service), *Oracle Service 1:B* exchanges *<$PK_{B}$, $PK_{2}$>* with *Oracle Service 2*, and *Oracle Service 2* has the public key $PK_{X}$ of each Oracle Service 1, where x∈X,X={A,B}. Therefore, each oracle service can use the other public key to encrypt data when interacting with each other, but *Oracle Service 2* and *Oracle Service 1* only involve cross-chain requests and data transfer of the specified service, not the target data. Due to the normal workflow and strength of the public oracle service, encrypted target data are returned to *Oracle Service 1* instead of *Oracle Service 2*.

According to the inter-chain interaction rules, *Oracle Service 1:A* generates *encryptToTRes* using a $PK_{2}$ encryption cypher to communicate *reqInfo* (Equation (3)) with *Oracle Service 2* and *Oracle Service 2* decrypts and generates *encryptToXRes* with a $PK_{X}$ encryption cypher of the public key of the specified service and sends it according to the predefined interaction format stitching and combination *transInfo* (Equation (4)) to the target service. Here, *X* refers to *Oracle Service 1:B*. The symmetric key, *SK*, for the password, *pwd*, is generated, which is used to selectively encrypt the important private data in the acquired data in subsequent interactions between *Oracle Service 1:B* and *Oracle Service 1:A*. Furthermore, the encrypted data, *SK<data>*, is delivered. Since *SK* is specified by the cross-chain requesting *Oracle Service 1:A*, the target data can be decrypted, thus ensuring the security of the data in the off-chain world and during transmission.

## 4. Results

To evaluate the feasibility and performance of our approach, we set up an experimental environment. We used the time delay as a key indicator to measure the performance of the cross-chain approach in the field. We evaluated the time delay required to complete the first secure cross-chain operation and to perform a series of secure cross-chain operations, representing the interaction time with no and existing connected channels, respectively. Under the same experimental conditions, the cross-chain time delay without using our mixed encryption method was compared to analyze the cost incurred by the introduction of this method.

### 4.1. Experimental Settings

Our experiments were configurated on the underlying consortium blockchain platform Fisco Bcos v2.8. The participating cross-chain consortium blockchains were deployed on the cloud server with Ubuntu Server 20.04 LTS OS, and the efficient cross-chain oracle was built on a host configured with AMD Ryzen7 5800H, Radeon Graphics 3.20GHz integrated graphics, and 16.0GB RAM.

### 4.2. Performance Analysis

We evaluated the performance of our approach using a recognized indicator in the field of cross-chain research, i.e., time latency. The time when the user initiates the request is the start time and when the destination data is returned, this is the end time. We focused on the total time of the closed loop of thecross-chain: *totalTime*. The whole cross-chain process contains several important stages, including *queryTime*, the consumed query time of collating data; *encryptDes*, the asymmetric key encryption and decryption time; and *encryptRes*, the time for symmetric keys to encrypt and decrypt data. They partly reflect the costs of mixed encryption methods.

**Figure 7:**
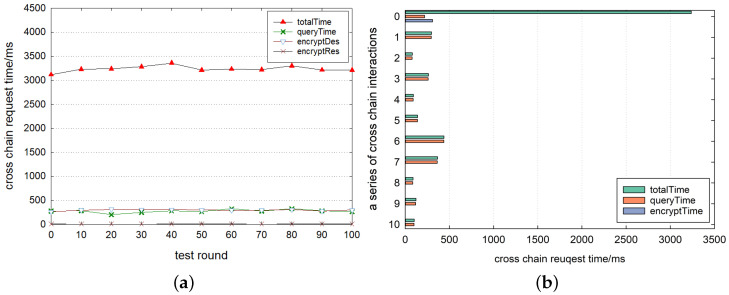
Cross-chain time latency. (**a**) First cross-chain request tests and (**b**) a series of cross-chain request tests.

Figure 7 shows the time latency for the complete oracle cross-chain method using our mixed encryption method with the off-chain transmission. It can be seen that the time consumption of the first connection for cross-chain interactions with another consortium blockchain is within 3000–3500 ms when the service starts and stops. *query_time* and *encrypt_des* are around 400 ms and *encrypt_res* continues to consume the least, this reflects that the cross-chain time latency is fully consistent with the response time of the human–computer interaction [20] and the cost of mixed encryption methods is small enough even without any connection and reusable resources. Furthermore, the overall results show a stable trend. As shown in Figure 7b, maybe for the first time, due to the multiplexing of connection and encryption resources, the *total_time* for cross-chain interactions is stable within 500 ms. This represents an extremely short cross-chain time latency, a performance rarely achieved in previous studies. Here, the *encryptTime* is also infinitesimally small. It reflects the time for *encryptDes* and *encryptRes* to be added together because they are small enough. At this point, *queryTime* dominates the time consumption. Combined with the actual cross-chain context, after the cross-chain oracle starts its service, most of the cross-chain transactions are not the first cross-chain, and the request time latency is within 500 ms, which presents the overall performance of the cross-chain interoperability method based on the blockchain oracle.

**Figure 8:**
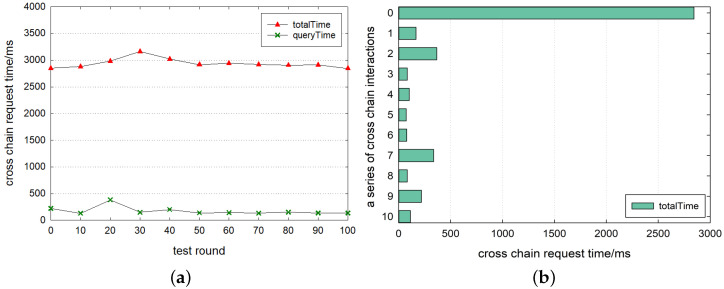
Unencrypted cross-chain time latency. (**a**) First cross-chain request tests and (**b**) a series of cross-chain request tests.

In the same experimental environment, we test the time consumption of the cross-chain process without the mixed encryption method. From the results in Figure 8a, it can be seen that the time latency between consortium blockchains for the first cross-chain communication is stable after around 3000 ms and in Figure 8b, the time latency of non-first cross-chain requests is stable within 500 ms. Analyzing Figure 7 and Figure 8, we can see that the impact of off-chain transmission mixed encryption on the first cross-chain communication is less than 300 ms. From the *encryptTime* in Figure 7b, the non-first cross-chain requests are almost unaffected. In the actual cross-chain application, the requests that are not the first time are the whole performance, and the influence caused by the mixed encryption method of off-chain transmission is extremely small. Therefore, the security of off-chain transmission can be completely guaranteed with such a small time cost, and we have achieved security at a negligible cost.

## 5. Discussion

To further demonstrate that our method can achieve high efficiency and low latency cross-chain data interoperability between consortium blockchains, we collated the effects of different cross-chain methods in the field. The results are shown in Table 1. In paper [9], focusing on the shortcomings of cross-chain technology in the storage of massive data, a cross-chain scheme of agent networks was proposed. In this scheme, agent nodes were elected to build the cross-chain network. Experimental results showed that the average cross-chain delay of this scheme was 0.375 s. However, because only a simulation experiment was performed in this paper, and no actual inter-chain environment was built, the experimental result only reflected the time of information transmission and it could not evaluate the time of connection establishment and inter-chain interactions in the virtual blockchain environment, so it was difficult to reflect the application effect of this method in the blockchain. Wu et al. [12] proposed a cross-chain communication framework based on the cyclic committee rotation mechanism. Different blockchains were connected through committees for message-oriented verification to improve the cross-chain access speed. The experiment was still implemented in the simulation environment, and an average cross-chain delay effect of 5 s was achieved. In the experiment, the election of committee members consumed most of the time. The cross-chain workflow model proposed in the article [11] defined the cross-chain process as a cross-chain workflow, which abstracted the cross-chain process with similar characteristics and realized the management of cross-chain workflow with the relay chain as the centre. According to the experimental results obtained in this paper, because of the involvement of the relay chain, the burden of inter-chain interactions was increased and the average cross-chain time was 10.47 s. The management system based on cross-chain switching designed by Tan et al. [14] introduced public key encryption with a keyword search to improve the scalability of blockchain applications in vehicular ad hoc networks. An average cross-chain exchange performance of 10.5 s was obtained in the experiment built in an actual blockchain environment. In addition, the security of inter-chain interactions was considered, and part of the security was guaranteed by public key encryption. The transaction-based asynchronous cross-chain exchange model in article [10] had embedded control conditions to determine whether asset transfer occurred. Control conditions were used to specify paired transactions and find the cross-chain transactions that met the conditions. The experimental results showed that the average cross-chain time was 12 s, which was relatively long. An analysis of the scheme showed that the scheme changed the underlying transaction structure and consumed a long time in the process of embedding control conditions. Qiao et al. [13] proposed the cross-chain communication method of node topology, which defined the construction rules of node identity path proof and dynamically constructed and verified the cross-chain transaction path proof. The experimental results showed that the average communication time between nodes of different consortium blockchains in this method was about 13 s. Inter-chain interactions were promoted by nodes playing different roles in the consortium blockchains.

The average time latency achieved by the current cross-chain approach is between 0.375 and 13 s across different blockchain platforms and experimental settings. Among them, the experiment performed in 0.375 s was not established in an actual blockchain environment, so the costs of connections and the performance of on-chain queries could not be realistically evaluated. However, our experiments were completely built in the actual inter-chain interaction context. The overall performance of our approach is within 0.5 s, and a few cross-chain performances are within 3.5 s, all within the effect range achieved by the current cross-chain solutions and in a relatively advanced position. As discussed in some current cross-chain schemes, due to the diversity of underlying blockchain technology and differences in cross-chain scenes, we need help to compare experimental effects by focusing on control variables in the same platform and experimental environment. However, among the existing excellent schemes and experimental effects of different underlying platforms, We have demonstrated that the proposed CCIO can achieve high efficiency and low delay cross-chain interaction performance, and the experimental results are also at the forefront of the current solutions.

We optimized and improved various oracle patterns to meet the cross-chain request prompt push and active pull of request results. After initiating the cross-chain request, the program will automatically complete the cross-chain process in time without active human interference. To meet cross-chain timeliness, we have improved the push-based outbound oracle to collect and process cross-chain requests. Once a cross-chain request is found, it is immediately outbound to enter the subsequent process. At the same time, we optimized and combined the push-based inbound oracle and pull-based outbound oracle, which immediately pull the result out of the source consortium blockchain and push it into the request consortium blockchain. In the process, the public oracle service assumes the role of validation, collection of service information, and designation of service, effectively indicating each process’s next step and direction. Although some schemes have introduced public key encryption to ensure the security of the cross-chain process, the public key can only encrypt a limited amount of data. We improve the use of a symmetric key to encrypt data and the asymmetric key to encrypt symmetric cryptography, to eliminate the size limit of encryptable data and ensure the security of the interaction process. This is how CCIO realizes such a high efficiency and low delay data interaction effect in multiple real consortium blockchain environments based on the security guarantee.

In the future, we will also work to study the issues of consortium blockchain interoperability and make CCIO more practical and easy to validate, as well as universal. We will select a typical cross-chain scenario, build an interactive environment between consortium blockchains, apply our proposed CCIO to this scenario, and demonstrate its use in a case study. We will continue to test the CCIO method that has achieved better performance under an ideal experimental environment and its performance effect in actual application scenarios. In the application environment, we will focus on concurrency and security to ensure low delay to achieve the same performance as in the experimental environment. Efforts to continuously optimize the method to minimize the additional time delays and costs associated with intervening in real-world scenario interactions will then be refined to make the method more practical.

## 6. Conclusions

In this paper, we propose CCIO, which successfully improves the efficiency of inter-chain interoperability while ensuring standardized and secure interactions. We first analyze the inter-chain data interaction architecture and interaction flow applied to the consortium blockchains, then we discuss the details of the method to regulate the interaction flow by predefined inter-chain interaction rules. We novelly improve three oracle patterns to build an efficient cross-chain oracle and introduce the principle and implementation details of combining a mixed encryption method of symmetric and asymmetric keys to ensure the security of off-chain transmissions. Finally, the experimental results of inter-chain interactions based on an actual consortium blockchain show CCIO can achieve efficient, low-latency, and secure bidirectional data interaction across consortium blockchains.

In the future, we will still devote ourselves to studying the problems in data interactions and take courageous steps towards selecting a typical cross-chain interactive scenario and applying CCIO to the actual inter-chain application in consortium blockchains. Furthermore, new attempts will be made to minimize the loss in efficiency while ensuring the integrity and security of interoperability.

## Figures and Tables

**Table 1:** Comparison of results of different platforms and cross-chain solutions.

Experimental Environment	Max/s	Min/s	Mean/s
Simulation [9] (Ubuntu 20.04.1, AMD Ryzen5 4600H			
CPU with Radeon Graphics 3.00 GHz)	0.6	0.15	0.375
**Fisco Bcos v2.8 (Ubuntu Server 20.04, AMD Ryzen7 5800H**			
**with Radeon Graphics 3.20 GHz, 16 GB memory)**	**3.41**	**0.076**	**0.418**
Simulation [12] (Intel core i5-4590, 3.30 GHz, 8.00 GB RAM)	7	3	5
Cosmos sdk v0.42 to build blockchains [11]	11	9.94	10.47
Ethereum, truffle, Ganache [14] (Intel core i7			
2.6 Ghz CPU,16G RAM, Radeon Pro 555X 4GB)	14	7	10.5
Ethereum [10] (DELL Power Edge T130,			
4 IntelXeon CPU E3-1220 v5 @3.00GHz 32 GB memory)	17	7	12
Consortium based on Ethereum [13], (IntelXeonE5 CPU			
64 GB memory, Ubuntu64 os)	15	11	13

## Data Availability

The data presented in this study are available on request from the corresponding author.

## References

[1] Touloupou M., Christodoulou K., Inglezakis A., Iosif E., Themistocleous M. Towards a Framework for Understanding the Performance of Blockchains. Proceedings of the 2021 3rd Conference on Blockchain Research & Applications for Innovative Networks and Services (BRAINS).

[2] Yang D., Long C., Xu H., Peng S. A Review on Scalability of Blockchain. Proceedings of the ICBCT’20: The 2nd International Conference on Blockchain Technology.

[3] Jin H., Dai X., Xiao J. Towards a Novel Architecture for Enabling Interoperability amongst Multiple Blockchains. Proceedings of the 2018 IEEE 38th International Conference on Distributed Computing Systems (ICDCS).

[4] Gordon W.J., Catalini C. (2018). Blockchain Technology for Healthcare: Facilitating the Transition to Patient-Driven Interoperability. Comput. Struct. Biotechnol. J..

[5] Li F., Li Z., Zhao H. (2019). Research on the Progress in Cross-chain Technology of Blockchains. J. Softw..

[6] Wang Y., Liu H., Wang J., Wang S. Efficient Data Interaction of Blockchain Smart Contract with Oracle Mechanism. Proceedings of the 2020 IEEE 9th Joint International Information Technology and Artificial Intelligence Conference (ITAIC).

[7] Gao Z., Li H., Xiao K., Wang Q. Cross-chain Oracle Based Data Migration Mechanism in Heterogeneous Blockchains. Proceedings of the 2020 IEEE 40th International Conference on Distributed Computing Systems (ICDCS).

[8] Chang J., Ni J., Xiao J., Dai X., Jin H. (2022). SynergyChain: A Multichain-Based Data-Sharing Framework with Hierarchical Access Control. IEEE Internet Things J..

[9] Wang W., Wang J., Wang Z., Liu Y. A Cross-Chain Solutions Based On Proxy Network. In Proceedings of the 2021 18th International Computer Conference on Wavelet Active Media Technology and Information Processing (ICCWAMTIP).

[10] Su H., Guo B., Lu A.J. (2022). Cross-chain Exchange by Transaction Dependence with Conditional Transaction. Soft Comput..

[11] Wu X. Cross-Chain Workflow Model Based on Trusted Relay. Proceedings of the ACM Turing Award Celebration Conference-China (ACM TURC 2021).

[12] Wu Z., Xiao Y., Zhou E., Pei Q., Wang Q. A Solution to Data Accessibility Across Heterogeneous Blockchains. Proceedings of the 2020 IEEE 26th International Conference on Parallel and Distributed Systems (ICPADS).

[13] Qiao R., Luo X.Y., Zhu S.F., Liu A.D., Yan X.Q., Wang Q.X. (2020). Dynamic Autonomous Cross Consortium Chain Mechanism in e-Healthcare. IEEE J. Biomed. Health Inform..

[14] Tan C., Bei S., Jing Z., Xiong N.N. (2021). An Atomic Cross-Chain Swap-Based Management System in Vehicular Ad Hoc Networks. Wirel. Commun. Mob. Comput..

[15] Albreiki H., Rehman M.H.U., Salah K., Svetinovic D. (2020). Trustworthy Blockchain Oracles: Review, Comparison, and Open Research Challenges. IEEE Access.

[16] Mühlberger R., Bachhofner S., Ferrer E.C., Ciccio C.D., Weber I., Whrer M., Zdun U. (2020). Foundational Oracle Patterns: Connecting Blockchain to the Off-chain World. Lecture Notes in Business Information Processing.

[17] Woo S., Song J., Park S. (2020). A Distributed Oracle Using Intel SGX for Blockchain-Based IoT Applications. Sensors.

[18] Ma L., Kaneko K., Sharma S., Sakurai K. Reliable Decentralized Oracle with Mechanisms for Verification and Disputation. Proceedings of the 2019 Seventh International Symposium on Computing and Networking Workshops (CANDARW).

[19] Heiss J., Eberhardt J., Tai S. From Oracles to Trustworthy Data On-Chaining Systems. Proceedings of the 2019 IEEE International Conference on Blockchain (Blockchain).

[20] Dabrowski J., Munson E.V. (2011). 40years of searching for the best computer system response time. Interact. Comput..

